# Modified Tumor Classification With Inclusion of Tumor Characteristics Improves Discrimination and Prediction Accuracy in Oral and Hypopharyngeal Cancer Patients Who Underwent Surgery

**DOI:** 10.1097/MD.0000000000001114

**Published:** 2015-07-13

**Authors:** Ching-Chih Lee, Hsu-Chueh Ho, Yu-Chieh Su, Chia-Hui Yu, Ching-Chieh Yang

**Affiliations:** From the Department of Otolaryngology, Dalin Tzu Chi Hospital, Buddhist Tzu Chi Medical Foundation, Chiayi, Taiwan (C-CL, H-CH); School of Medicine, Tzu Chi University, Hualian, Taiwan (C-CL, H-CH, Y-CS); Division of Hematology-Oncology, Department of Internal Medicine, Dalin Tzu Chi Hospital, Buddhist Tzu Chi Medical Foundation, Chiayi, Taiwan (Y-CS); Department of Medical Research, Dalin Tzu Chi Hospital, Buddhist Tzu Chi Medical Foundation, Chiayi, Taiwan (C-HY); Department of Radiation Oncology, Chi-Mei Medical Center, Tainan, Taiwan (C-CY); and Institute of Biomedical Sciences, National Sun Yat-Sen University, Kaohsiung, Taiwan (C-CY).

## Abstract

Several histopathological characteristics have a significant prognostic impact on recurrence and survival rates in head and neck squamous cell carcinoma (HNSCC). We conducted a retrospective study on patients with HNSCC to compare traditional pathological T (pT) classification to a new T classification system that incorporates these histopathological characteristics.

Newly diagnosed patients with HNSCC (n = 349) post major surgery were identified from the cancer registry database between 2004 and 2013. The pT and new T classification systems were compared with respect to recurrence-free survival (RFS), disease-specific survival (DSS), and survival rates using the Cox proportional hazards model with adjustments. The discriminatory ability of these 2 classification systems was evaluated using the adjusted hazard ratio (HR) and Akaike information criterion (AIC) in a multivariate regression model. The prediction accuracy was assessed using Harrell's C-statistic.

The new T classification, which incorporated tumor size, extent, and location with histopathological features had better discriminatory ability and monotonicity of gradients than did pT classification. The new T4 classification yielded a higher adjusted HR in RFS (HR, 4.11; 95% confidence interval [CI], 7.75–9.65) and in DSS (HR, 4.39; 95% CI, 1.6–12.03), and a lower AIC in recurrence (927 vs 969) and survival rates (791 vs 833).

The new T classification system had better discriminatory ability in RFS and DSS compared with the routinely used American Joint Committee on Cancer (AJCC) pT classification system. Therefore, this new T classification system, which includes tumor size, location, extent, and histopathological features, could be used as an alternative to AJCC pT classification for patients with HNSCC.

## INTRODUCTION

Head and neck squamous cell carcinoma (HNSCC) is among the 10 most common forms of cancer, with a rising incidence in Western and Asian countries.^1,2^ Despite advances in clinical treatment, long-term outcomes of patients with HNSCC have not improved significantly in the last decades.^3,4^ Improvement and adjustment of the present TNM staging system may be necessary to identify high-risk groups.

The American Joint Committee on Cancer (AJCC) TNM classification system is widely applied to mucosal cancers of the head and neck. Within this system, the present pathological T (pT) classification for HNSCC is based on tumor size, extension, anatomic location, and cranial nerve involvement.^5^ Despite consideration of clinical prognostic factors such as tumor size or stage, prediction of the clinical outcome of HNSCC is difficult. Patients who have a small tumor may still have a poor outcome.^6^ Evaluation of the histopathological characteristics of resected tumor specimens plays an important role in the diagnostic process and prediction of patient outcomes. Multiple histopathological factors predicting survival have been identified in the literature, including tumor thickness, grade, pathological nodal classification, extracapsular spread, margin status, and perineural invasion (PNI).^7^ Thus, incorporation of these additional prognostic factors may be helpful for refinement of the classification system to improve its prediction accuracy.

Several prediction models have been proposed. Among oral SCCs, poor differentiation was associated with neck metastasis, extracapsular spread, PNI, neck recurrence, and distant metastasis.^8,9^ However, the differentiation grade was not regarded as a risk factor in determining adjuvant therapy in the latest NCCN guidelines.^10^ Brandwein-Gensler et al validated the prognostic influence of histological findings, such as PNI, lymphocyte infiltrate at the interface, and worst pattern of tumor invasion in head and neck cancer (HNC).^11–13^ The depth of invasion, tumor budding, and worst pattern of invasion were independent prognostic factors for T1/T2 N0M0 tongue cancer.^14^ Vascular invasion along with tumor dimension, extracapsular spread, and comorbidity had better discrimination for overall survival in tongue cancer.^7^ In cutaneous SCC, the 7th edition AJCC T classification incorporated histological findings with tumor size and location.^15^ However, the combination of tumor size, location, and histological findings in this classification system have not been well developed for HNCs.

The tumor size, extension, and location remain important prognostic factors. Instead of abandoning the traditional tumor size and location, we devised a new classification system by incorporating histopathological features into the AJCC T classification to produce a new T classification similar to that used in cutaneous SCC AJCC staging.^15^ The aim of this study is to compare this new T classification to the current AJCC pT classification with respect to survival outcome prediction in patients with HNSCC using data from a single institution.

## MATERIALS AND METHODS

### Ethics Statement

This study was approved by the Institutional Review Board of Buddhist Dalin Tzu Chi General Hospital in Taiwan. Review board requirements for written informed consent were waived because all personal identifying information was removed from the dataset before analysis.

### Patient Demographics and Database

These data for this study were collected from the Cancer Registry Dataset from the Buddhist Dalin Tzu Chi General Hospital Cancer Center from 2004 to 2013. The electronic medical records and cancer registry dataset were retrospectively reviewed in detail. Cancer diagnoses and Elixhauser comorbid scores representing the conditions were recorded using codes of the International Classification of Diseases, 9th revision (ICD-9).^16^ Patients (n = 349) with a histologic diagnosis of primary resectable HNSCC after major surgery as the main treatment with or without adjuvant therapy were enrolled in this study. Patents with synchronous cancer, a history of cancer, or chemoradiotherapy as initial treatment were excluded. The cancer registry dataset included the date of diagnosis, site of the primary tumor, age, gender, margin status (positive or negative), degree of differentiation (well, moderate, or poor), retrieved lymph node, status of PNI, vascular permeation, lymphatic invasion, extra-capsular spread, chemotherapy, radiotherapy, cause of death, clinical TNM stage, and pathological TNM stage. HNSCC was staged according to the AJCC stage classification system, modified in 2009 (7th edition). The clinical endpoint was the 3-year disease-specific survival (DSS) rate and recurrence-free survival (RFS) rate. Death or recurrence (local/regional/distant metastasis) from cancer was recorded as an event in DSS rate or RFS rate.

### Optimal Category for New T Classification

We propose a revised category for a new T-staging system that was determined as follows:

1. We divided patients with HNSCC by AJCC T classification merged with a number of high-risk features, ranging from 0 to 2.
2. The 3-year RFS and DSS rates for each combination of AJCC T category and risk features were estimated with Kaplan–Meier method. Patients with HNSCC with similar survival rates were grouped together to form 4 categories, new T1–T4.
3. Subgroups with <10 patients with HNC were not included due to unstable estimation of survival rates.

### Statistical Analysis

All statistical operations were performed using SPSS (version 15, SPSS Inc., Chicago, IL). Cumulative 3-year RFS and DSS rates for the AJCC pT and new T classification systems were analyzed using the Kaplan–Meier method and compared using the log-rank test. Survival curves were measured from the time of diagnosis using disease-specific mortality as the primary event variable and RFS as the secondary outcome. The Cox proportional hazards regression model was used to compare outcomes of different T categories after adjusting for patient characteristics (age, gender, and comorbidity condition), pathological N (pN) status, and treatment modalities (radiotherapy and chemotherapy). We then identified independent risk factors for DSS and recurrence survival rates. Using a multivariate Cox hazard regression model with stepwise selection, we determined that poor differentiation, PNI, vascular permeation, and lymphatic invasion were significant risk factors for RFS and DSS.

The discriminatory ability and monotonicity of gradient evaluation for AJCC T classification and new T classification were explored using the linear trend Chi-squared test. The Akaike information criterion (AIC) for each regression model was also used to measure its discrimination.^17^ In multivariate analysis, we compared the adjusted hazard ratio (HR) and AIC for each regression model.^17^ A higher HR indicates a better system. In addition, a smaller AIC indicates a more discriminatory system. We used Harrell's C-statistic to assess the accuracy of the T classification system or regression model. The Harrell's C-statistic indicates the T classification or model prediction as follows: 0.5, equal to chance; 0.7–0.8, acceptable; 0.8–0.9, excellent; and 0.9–1, outstanding. A 2-sided *P* value (*P* < 0.05) was considered statistically significant.

## RESULTS

The demographic characteristics of the cohort are summarized in Table 1. Included in this study are 349 patients who underwent major surgery with curative intent for HNSCC with or without adjuvant therapy, with a mean follow-up of 23 ± 11 months. Patients were mostly men (n = 331; 94.8%), with 18 women (5.2%). The collective mean age was 54 ± 11 years. Most of the patients with HNSCC had oral cancer (n = 281; 80.5%); 68 (19.5%) had cancers in other sites such as the oropharynx or hyopharynx. For the whole group, the overall 3-year DSS was 71.1% and the 3-year RFS was 70.9%. Advanced pT classification was significantly associated with poor differentiation, lymphatic permeation, vascular permeation, and PNI (Table 2).

**TABLE 1:**
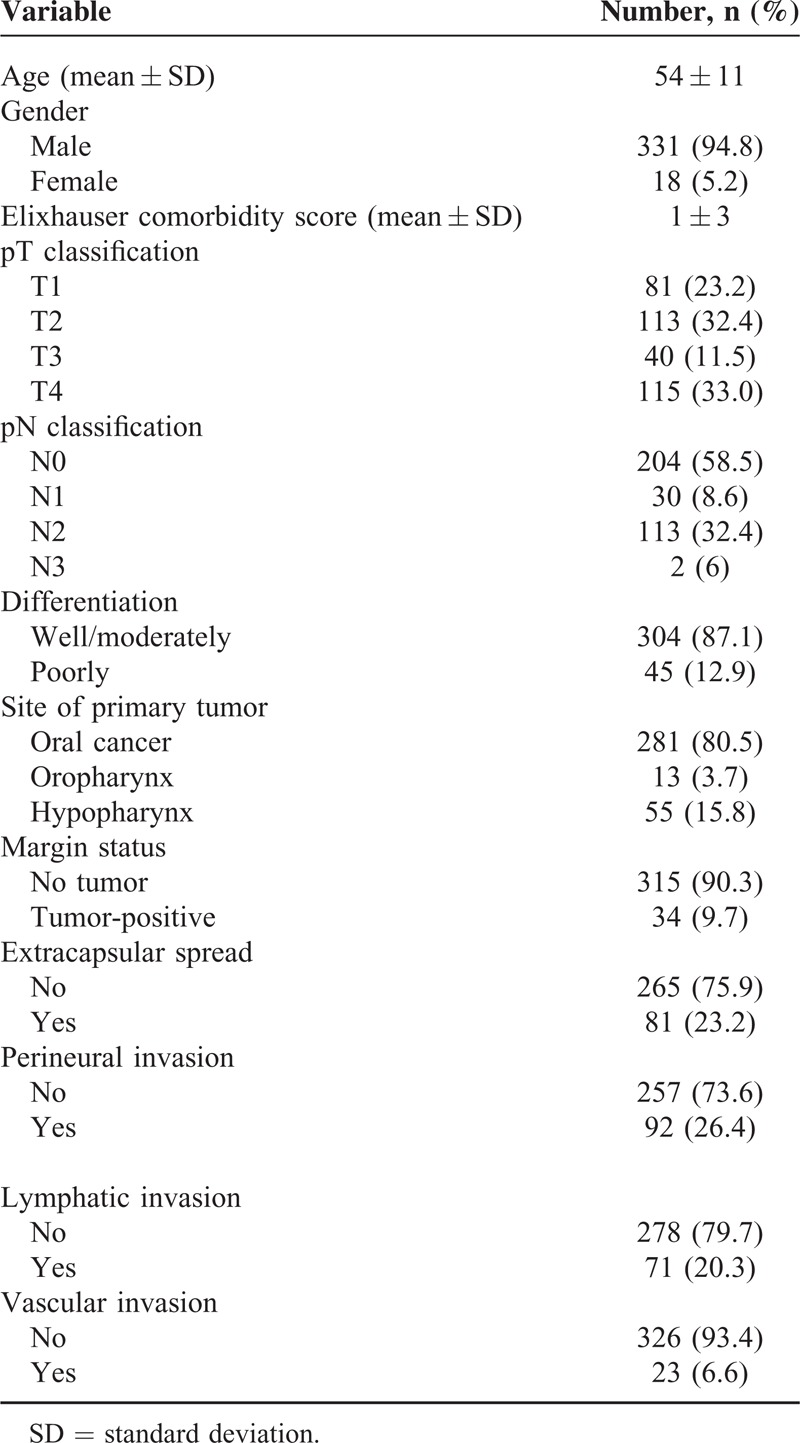
Demographic and Clinical Characteristics of Study Patients, n = 349

**TABLE 2:**
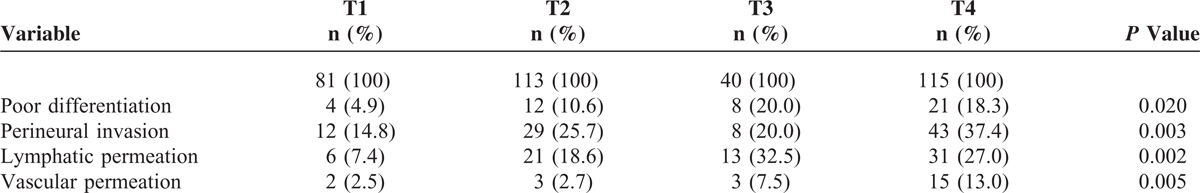
Distribution of Histological Features and pT Classification

**FIGURE 1:**
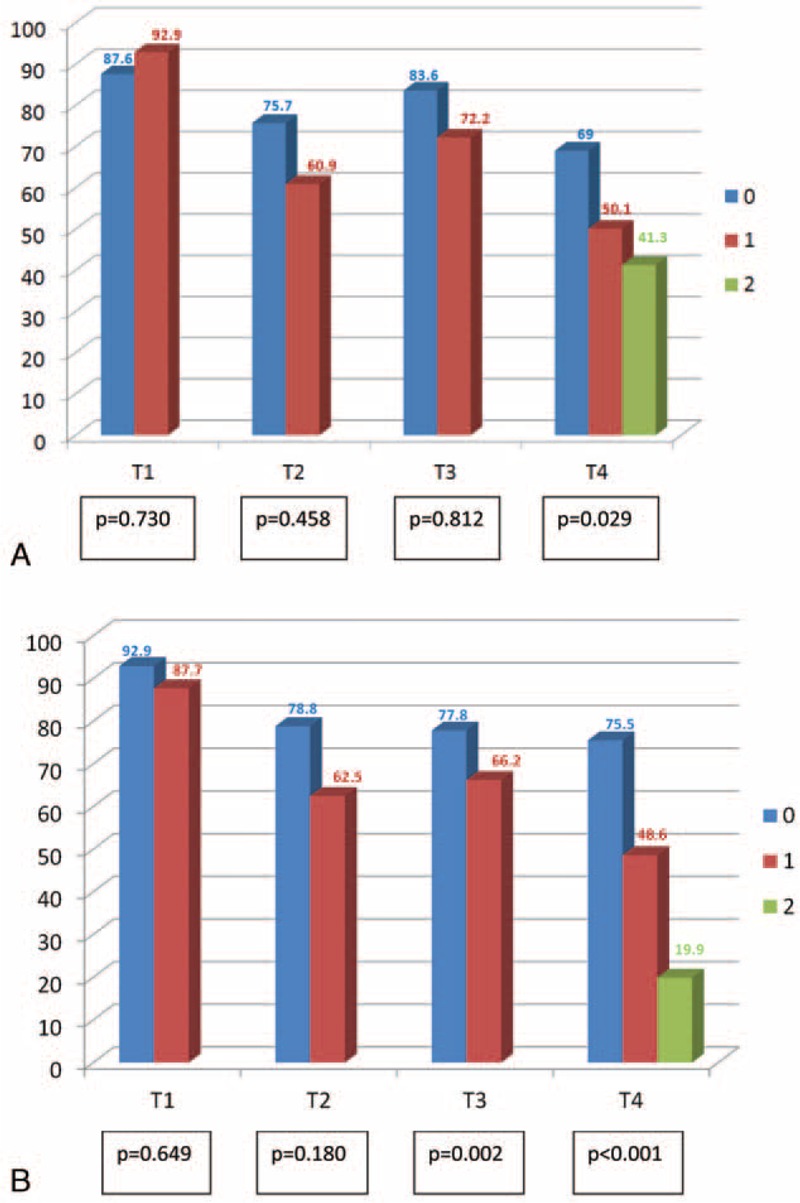
(A) Impact of high-risk features on 3-year RFS by pT classification. (B) Impact of high-risk features on 3-year DSS by pT classification. DSS = disease-specific survival; RFS = recurrence-free survival.

We further explored the impact of histological features on clinical outcomes. For RFS rates, poor differentiation was a prognosticator (HR, 1.8; 95% confidence interval [CI], 1.06–3.04). For DSS rates, poor differentiation (HR, 3.16; 95% CI, 1.87–5.33) and perineural/vascular/lymphatic invasion (HR, 1.79; 95% CI, 1.12–2.86) were independent prognostic factors (Table 3). Figure 1 shows the impact of high-risk features on the RFS and DSS in different pT classifications. Patients with pT4 HNSCC that had more risk features incurred grave RFS (Figure 1A). For DSS, histological features had a significant impact on survival rates for patients with pT3 and pT4 (Figure 1B).

**TABLE 3:**
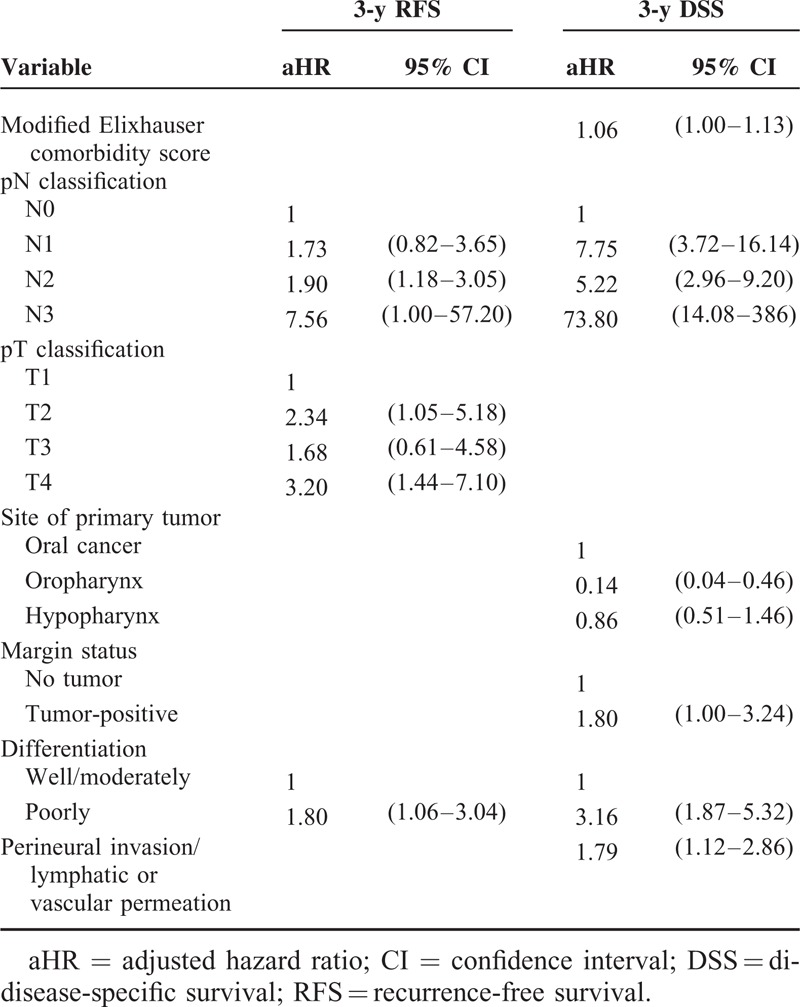
Multivariate Analysis for Independent Variables for 3-Year Recurrence-Free Survival and Disease-Specific Survival Rate With Backward Stepwise Selection

The 3-year RFS and DSS for different combinations of AJCC pT and number of high-risk features are summarized in Table 4. As described in the methods section, subgroups with <10 patients with HNSCC were not included in further analysis, eliminating subgroup AJCC pT1 (with 2 high-risk features) as well as T2 and T3 (each with 2 high-risk features). Although patients with pT3 with 1 risk factor had better 3-year RFS than patients with pT4 without risk features, a higher rate of distant metastasis was noted among patients with pT3 with 1 risk feature. We then grouped patients with pT3 with 1 risk factor into the new category 3. We proposed a new T classification as follows: T1, tumors <2 cm; T2, tumors >2 cm or advanced T without a high-risk feature; T3, tumors >2 cm with 1 high-risk feature; and T4, advanced T with at least 1 high-risk feature.

**TABLE 4:**
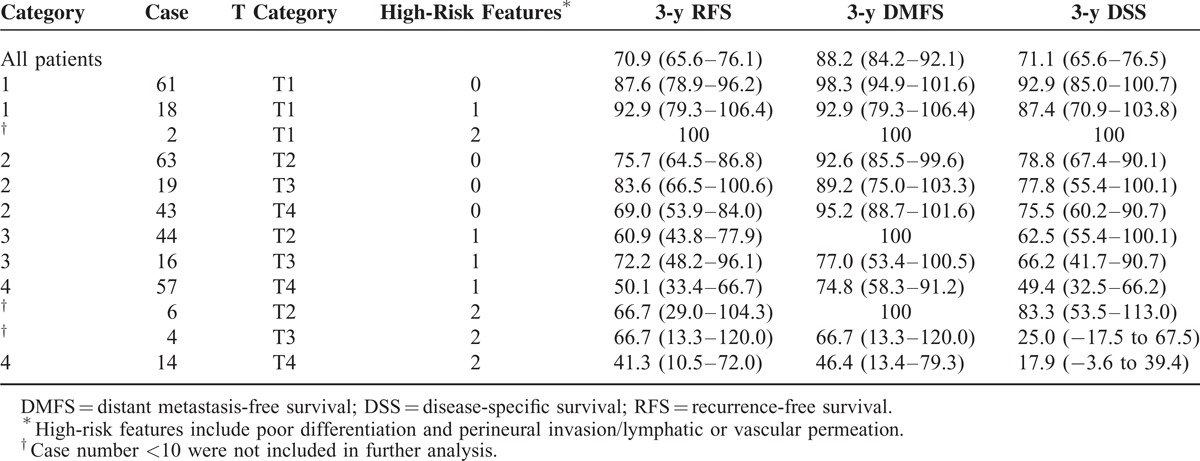
Summary of 3-Year Disease-Specific Survival Rates for Different Tumor and Characteristics Combinations

**FIGURE 2:**
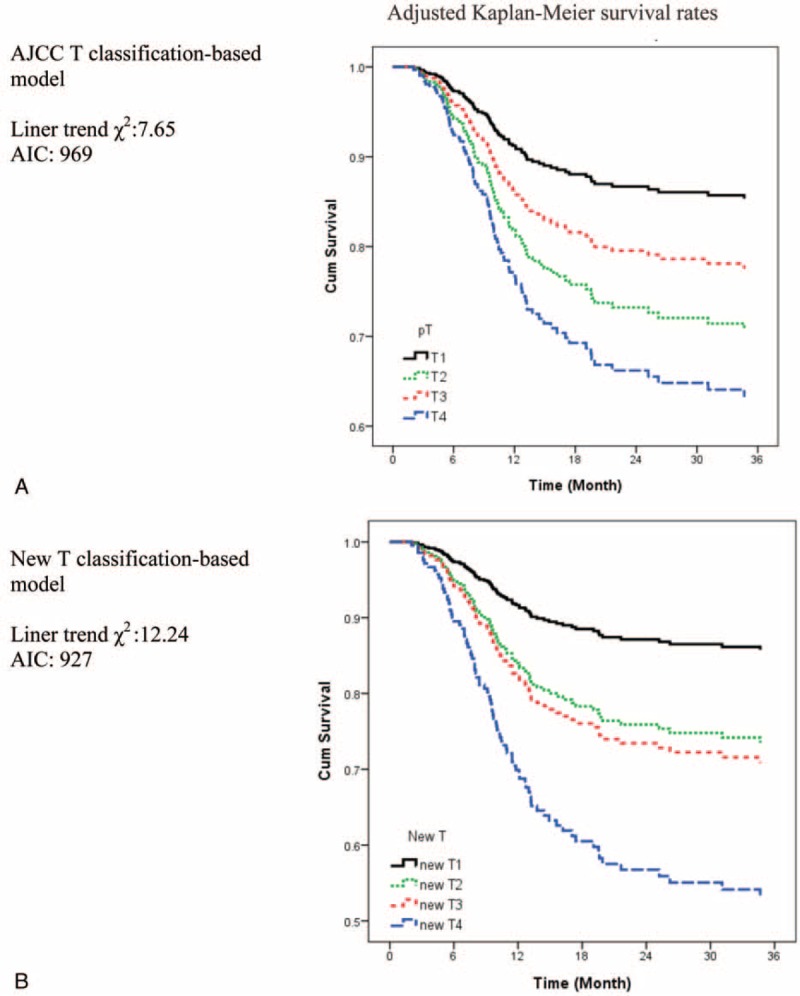
Adjusted recurrence-free survival curves for pT (A) and new T (B) classifications. (A) AJCC T classification-based model (liner trend χ^2^: 7.65; AIC: 969). (B) New T classification-based model (liner trend χ^2^: 12.24; AIC: 927). AIC = Akaike information criterion; AJCC = American Joint Committee on Cancer.

**FIGURE 3:**
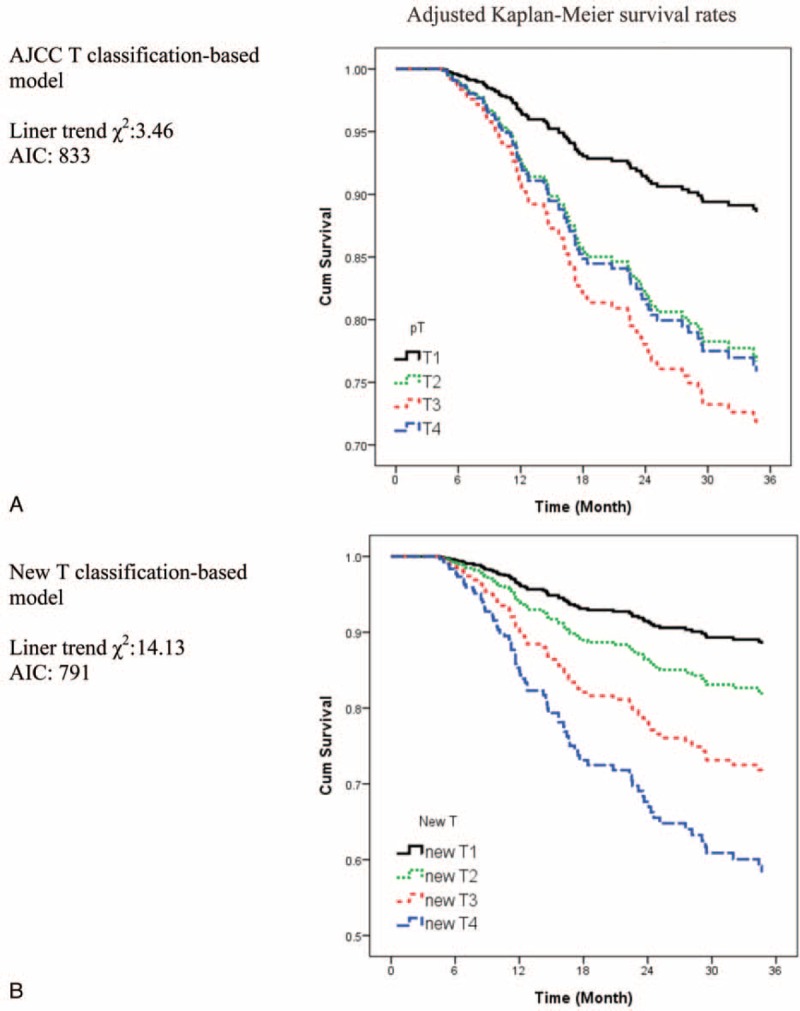
Adjusted disease-specific survival curves for pT (A) and new T (B) classifications. (A) AJCC T classification-based model (liner trend χ^2^: 3.46; AIC: 833). (B) New T classification-based model (liner trend χ^2^: 14.13; AIC: 791). AIC = Akaike information criterion; AJCC = American Joint Committee on Cancer.

We assessed the discriminatory ability of each T classification with adjusted HR and AIC values in a multivariate analysis model. The adjusted 3-year RFS for the pT category and new T category is shown in Figure 2. The new T classification had better discrimination for RFS with a smaller AIC (927 vs 969) and a higher linear trend Chi-square (12.24 vs 7.65). The new T4 had a higher adjusted HR (HR, 4.11; 95% CI, 1.75–9.69) (Table 5). Figure 3 shows the adjusted 3-year DSS for the pT and new T classification systems. After adjusting for risk features, the AJCC T classification was not an independent prognostic factor for 3-year DSS. The higher adjusted HR for the new T4 remained upon multivariate analysis (HR, 4.39; 95% CI, 1.6–12) (Table 6). The new T classification system had the lowest AIC value (791) and higher linear trend Chi-square (14.13).

**TABLE 5:**
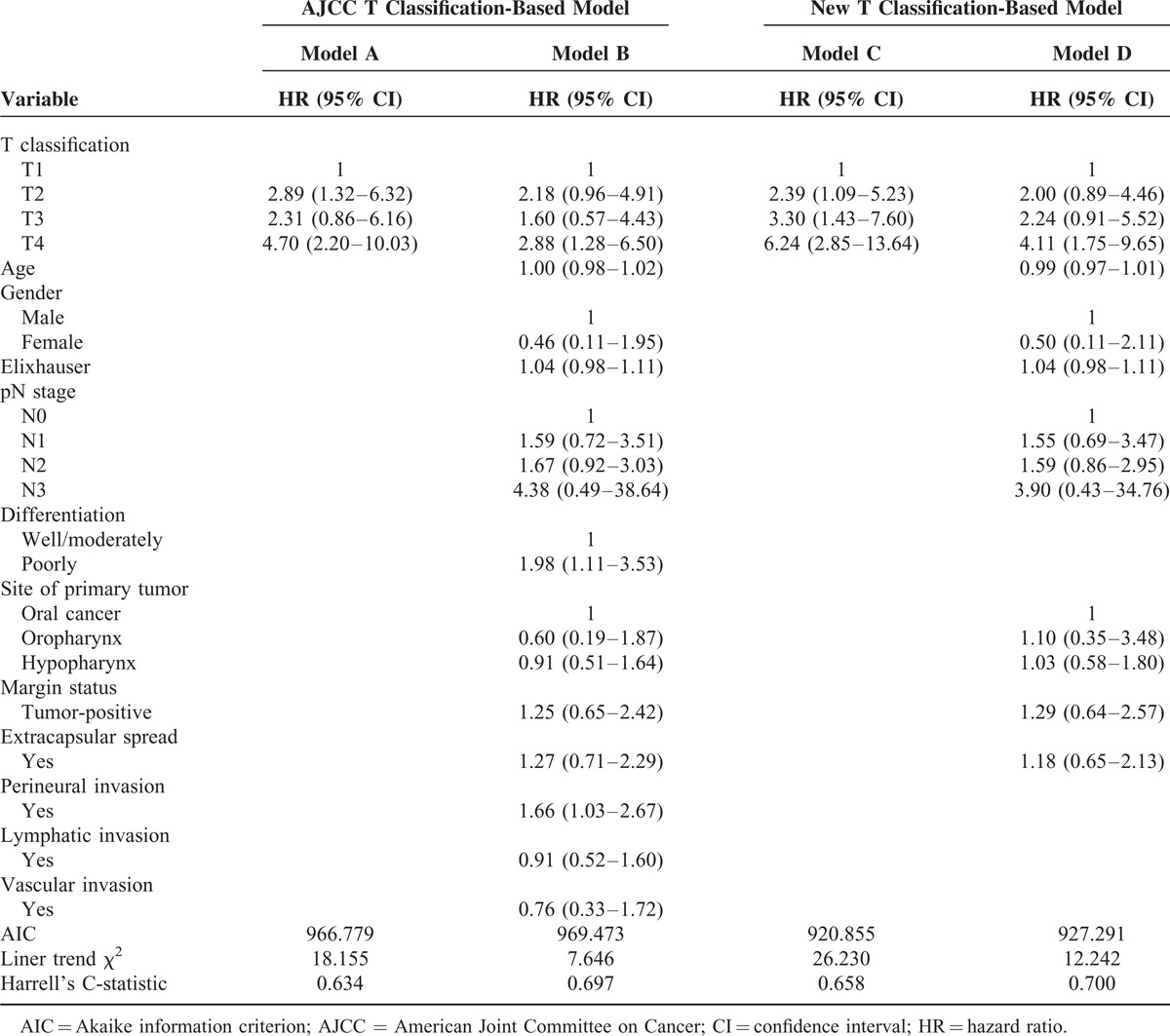
Adjusted Hazard Ratios for pT and New T Classification in 3-Year Recurrence-Free Survival Rates

**TABLE 6:**
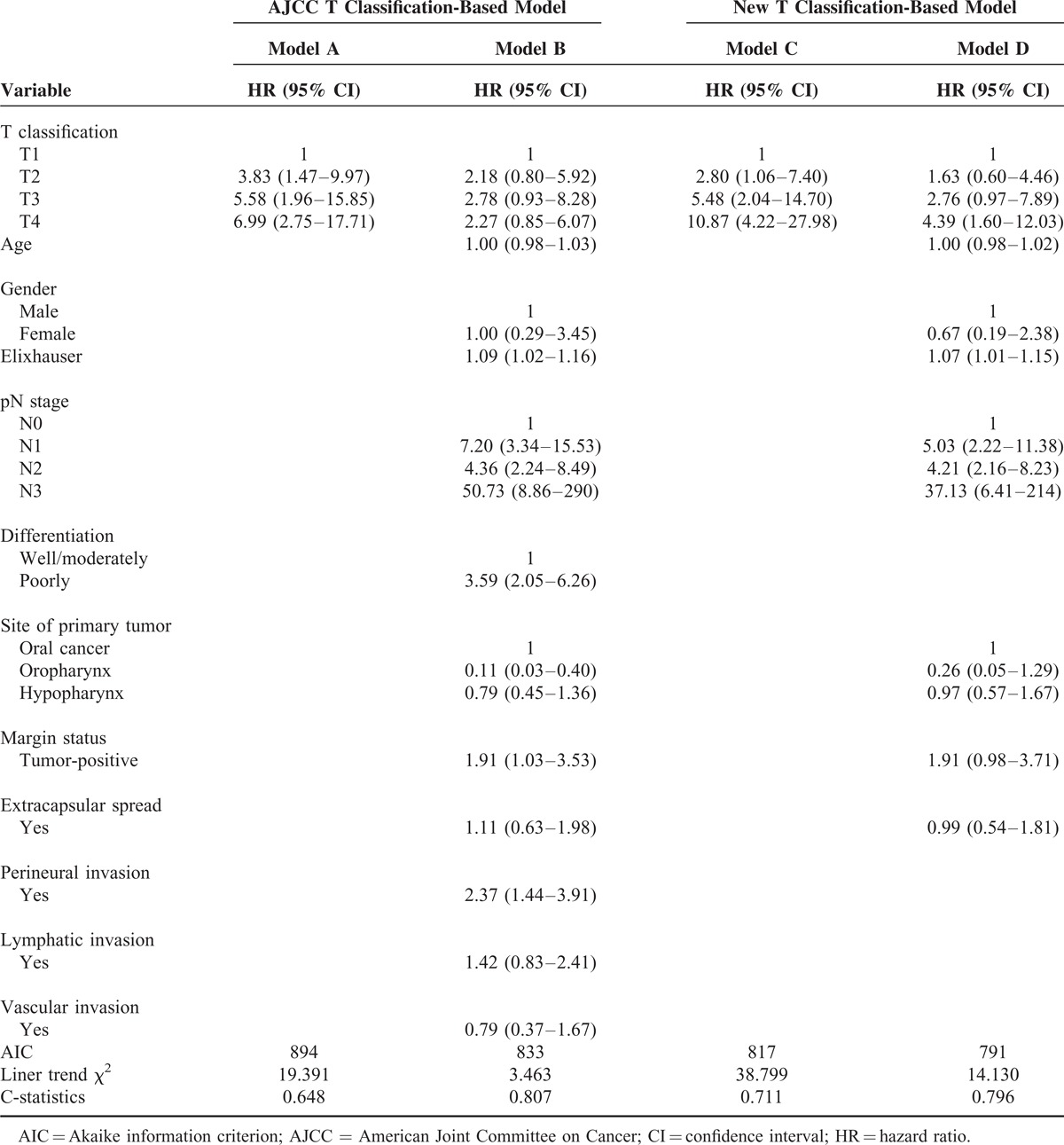
Adjusted Hazard Ratios for pT and New T Classification in 3-Year Disease-Specific Survival Rates

## DISCUSSION

This study proposed that the present pT classification system is deficient in estimating HNSCC survival outcomes and could be improved by incorporating histopathological high-risk features including poor differentiation, PNI, vascular permeation, and lymphatic invasion. For predicting survival of patients with HNSCC treated with curative surgery, the discriminatory ability, monotonicity of gradients, and prediction accuracy were statistically better with the new T classification. The inclusion of these high-risk features in the new T classification may help us to stratify a true high-risk group of patients who would benefit from adjuvant therapy.

Although there were more recurrences in oral cancer patients with T3–4 without adverse features, the survival rates between T2, T3, and T4 without adverse feature were not statistically different. Five of 10 patients among patients with pT4N0 with local or regional recurrences underwent salvage surgery with negative margin and adjuvant therapy; however, only 3 of 10 patients with local/regional recurrence were successfully salvaged. This could be one of the reasons why there was no statistical difference in survival rates between the pT2–4 without adverse features.

This study has several strengths. To our knowledge, this is the first study to merge the AJCC pT classification and histopathological risk features into a new T classification for HNC. Second, this new T classification improved the discrimination in multivariate regression model over that of the current AJCC pT classification. Several previous prediction models using histological features were only used to stratify the early-stage disease.^14,18^ Our study provided a new T classification that was suitable and convenient to use for both early- and advanced-stage disease. For patients with HNSCC undergoing surgical intervention, risk stratification that incorporates tumor size, location, and histopathological characteristics may better predict survival.

In recent years, the AJCC pT classification alone has been used to predict HNSCC survival and to decide on further treatment options.^19,20^ This classification does not take other clinically relevant factors such comorbidity, age, or the invasive profile of the malignancies into consideration. Support has increased for the use of other parameters in addition to the current TNM system to predict outcomes. In a study of 166 patients with tongue SCC, Okuyemi et al^7^ demonstrated that a clinicopathological model based on 4 clinicopathological variables (comorbidity, tumor dimension, extracapsular spread, and vascular invasion) provided better prediction of survival than did TNM staging alone (C-statistic, 0.736 vs 0.645, respectively). Comorbidity was the most significant factor associated with overall survival. Incorporation of tumor thickness into the TNM staging system has been shown to provide better survival outcome information in early oral cancer.^18^ We therefore studied a new T classification-nested model that substitutes high-risk features (poor differentiation, PNI, and lymphatic or vascular permeation) for tumor dimension (maximum dimension in centimeter) in the AJCC pT classification system. Our results reveal that comorbid conditions, pN classification, and margin status are not independent factors in multivariate analysis.

Poor tumor-differentiation status in patients after major surgery was the most significant pathological factor affecting survival in our analysis (HR, 3.16; 95% CI, 1.87–5.32), except for microscopically involved resection margins and/or extracapsular spread of the tumor from neck nodes, which has been recently proved as the most significant prognostic clinical factor for poor outcome.^21^ The overall prevalence of poor differentiation in our cohort was 12.9% (n = 45), a rate similar to those reported by other studies evaluating adjuvant therapy on survival of patients with HNSCC.^22,23^ The effect of tumor differentiation on HNSCC prognosis was first reported by Davis et al in 1985^24^ and has been investigated in further studies. A 20-year retrospective analysis of 87 cases of tonsillar SCC found that the major determinants of survival were tumor size and nodal status at presentation.^25^ The degree of tumor differentiation had no prognostic significance. In contrast, a more recent study reported by Larsen et al^9^ found tumor differentiation to be a strong prognostic factor for nodal metastasis, independent of other histological features. Patients with well and moderately differentiated tumors have significantly better nodal control and cause-specific survival than those with poorly differentiated tumors. Our results confirm the important role of tumor differentiation in survival and suggest the need to incorporate this factor into the current staging system.

Another known prognosticator of survival and local control in HNSCC is PNI. Fagan et al^26^ found significant differences in local recurrence (23% for PNI vs 9% for no PNI; *P* = 0.02) and disease-specific mortality (54% mortality for PNI vs 25% for no PNI; *P* < 0.001). Recently, Tarsitano et al^27^ also reported PNI as an independent predictor of local and regional failure in univariate and multivariate analysis. The invasive profile of the tumor (presence of lymphatic, perineural, and vascular invasion) also had an impact on survival in our study (HR, 1.79; 95% CI, 1.12–2.86). Although the idea of incorporating the invasive profile into the T classification is not new, we found that using these histological risk features improved survival prediction for surgically treated patients with HNSCC.

Comparison of the current AJCC pT to our new T classification revealed the impact of the new system on staging. In patients with HNSCC, the new T classification found significant differences in DSS and RFS in relation to the presence of histopathological risk features. Tumors <2 cm in diameter, categorized as the new T1, had the best prognosis. Because the adjusted survival for patients with T2 lesions and advanced T without high-risk lesions was very similar, these patients were grouped together in the new T2 category. T3 included tumors >2 cm in diameter with 1 high-risk feature. Finally, patients with advanced T and at least 1 high-risk feature had the worst prognosis and were grouped into the new T4 category. Compared to the current pT classification, the new T classification provides better heterogeneity in survival rates between categories (hazard discrimination) and better distribution of cases between categories (balance). Thus, according to the new T classification, patients who have early-stage pT1/2 HNSCC with high-risk features should receive adjuvant RT, possibly combined with chemotherapy, for greater tumor control. Prospective studies are necessary to prove the benefit of using adjuvant treatment in these patients.

This study has several limitations. First, the number of patients in some of the subgroups (AJCC pT1 with 1 high-risk feature and pT2/T3 with 2 high-risk features) was relatively small. The 3-year DSS was 83% (95% CI, 54–100) for pT2 with 2 high-risk features and 20% (95% CI, 0–55) for pT3 with 2 high-risk features. Survival rates were not included in the new T classification because estimates of 95% CI were unstable and wide. Large-scale prospective studies or those using a population-based cancer registry database with longer follow up may overcome these limitations. Second, although our series included patients with a variety of HNCs, 80.5% had oral cancer. Our results thus may not generalize to all HNCs. Third, the application of histopathological features to T classification requires a surgical specimen or adequate excision biopsy. Patients with HNSCC who do not undergo surgical intervention may not suitable for this classification. However, the new T classification improved discrimination between tumor features, which allowed us to identify true high-risk groups, such as patients with HNSCC with pT2–4 classification with at least 1 high-risk feature. Fourth, tumor depth, human *papillomavirus* (HPV) status, and p16 were not evaluated in this study. The rate of HPV among patients with oropharyngeal cancer in Taiwan is about 16%, which is lower than that reported in other series.^28,29^ Our series included 13 oropharyngeal cancer patients; thus, we estimate that about 2 of our patients may have HPV. This low number suggests that the lack of HPV infection status data may not influence our results. However, it should be performed at other institutions especially among the Western countries to generalize the applicability of this new T classification that incorporates high-risk HNSCC features. Finally, the depth of invasion has been regularly recorded in our pathological checklist since 2007. In order to recruit enough cases for analysis, this variable was not included in further analysis. It should be noted that this study did not include patients with very advanced local disease or unresectable tumors (AJCC T4b).

## CONCLUSION

In conclusion, the prognostic value of a new T classification that incorporates high-risk HNSCC features was confirmed. The new T classification system has better discriminatory and predictive ability than does the AJCC pT classification system. This new T classification could be used to further stratify patients with HNSCC and help us identify high-risk patients for adjuvant therapy.

## ACKNOWLEDGMENT

All authors thank the staff of the Center for Clinical Epidemiology and Biostatistics for the data preparation.
